# Bioactive Polyphenolic Compounds Showing Strong Antiviral Activities against Severe Acute Respiratory Syndrome Coronavirus 2

**DOI:** 10.3390/pathogens10060758

**Published:** 2021-06-15

**Authors:** Ahmed Kandeil, Ahmed Mostafa, Omnia Kutkat, Yassmin Moatasim, Ahmed A. Al-Karmalawy, Adel A. Rashad, Ahmed E. Kayed, Azza E. Kayed, Rabeh El-Shesheny, Ghazi Kayali, Mohamed A. Ali

**Affiliations:** 1 Center of Scientific Excellence for Influenza Viruses, National Research Centre, Giza 12622, Egypt; Ahmed.Kandeil@human-link.org (A.K.); ahmed_elsayed@daad-alumni.de (A.M.); Omnia.Abdelaziz@human-link.org (O.K.); Yasmin.Moatasim@human-link.org (Y.M.); Ahmed.Elsayed@human-link.org (A.E.K.); ra_eny@yahoo.com (R.E.-S.); 2 Department of Pharmaceutical Medicinal Chemistry, Faculty of Pharmacy, Horus University-Egypt, New Damietta 34518, Egypt; akarmalawy@horus.edu.eg; 3 Department of Biochemistry & Molecular Biology, Drexel University College of Medicine, Philadelphia, PA 19102, USA; aaa396@drexel.edu; 4 Radiation Biology Department, Atomic Energy Authority, Cairo 13759, Egypt; azzakaed60@gmail.com; 5 Department of Epidemiology, Human Genetics, and Environmental Sciences, University of Texas, Houston, TX 77030, USA; 6 Human Link, Jewellery & Gemplex, Dubai 48800, United Arab Emirates

**Keywords:** SARS-CoV-2, COVID-19, antiviral, curcumin, hesperidin, quercetin, molecular docking

## Abstract

Until now, there has been no direct evidence of the effectiveness of repurposed FDA-approved drugs against Severe Acute Respiratory Syndrome Coronavirus 2 (SARS-CoV-2) infections. Although curcumin, hesperidin, and quercetin have broad spectra of pharmacological properties, their antiviral activities against SARS-CoV-2 remain unclear. Our study aimed to assess the in vitro antiviral activities of curcumin, hesperidin, and quercetin against SARS-CoV-2 compared to hydroxychloroquine and determine their mode of action. In Vero E6 cells, these compounds significantly inhibited virus replication, mainly as virucidal agents primarily indicating their potential activity at the early stage of viral infection. To investigate the mechanism of action of the tested compounds, molecular docking studies were carried out against both SARS-CoV-2 spike (S) and main protease (Mpro) receptors. Collectively, the obtained in silico and in vitro findings suggest that the compounds could be promising SARS-CoV-2 Mpro inhibitors. We recommend further preclinical and clinical studies on the studied compounds to find a potential therapeutic targeting COVID-19 in the near future.

## 1. Introduction

Severe Acute Respiratory Syndrome Coronavirus-2 (SARS-CoV-2), a positive-sense single-stranded RNA virus, was first identified at the end of 2019 and has been declared by the World Health Organization (WHO) as a pandemic virus threatening global public health. The SARS-CoV-2 causes coronavirus disease (COVID-19), a respiratory illness ranging from asymptomatic to fatal disease. As of 17 March, 2021, COVID-19 was responsible for approximately 120 million confirmed human cases with more than 2.6 million human deaths [1]. Screening for effective antiviral agents for SARS-COV-2 based on natural or synthetic sources is desirable. Several research groups including ours screened several FDA-approved drugs as well as synthetic and natural compounds in a SARS-CoV-2 infection cell-based assay [2,3,4,5]. 

**Figure 1:**
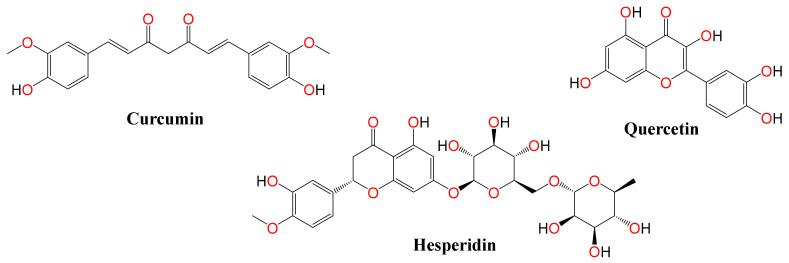
Chemical structures of the tested compounds (curcumin, quercetin, and hesperidin).

Based upon previous studies, curcumin, hesperidin, and quercetin have multi-biological activities and have been extensively used in traditional medicine (Figure 1). Curcumin is a bright yellow polyphenol compound produced as a major active ingredient in the rhizome of *Curcuma longa* [6]. It exists in an enolic form in organic solvents and in a keto form in water (Manolova et al., 2014). Curcumin exhibits varieties of therapeutic properties including antioxidant, anti-inflammatory, antiseptic, antitumor, analgesic, antimicrobial and antiviral activities [7,8]. Previous studies showed the antiviral efficacy of curcumin against RNA and DNA viruses via virucidal effects or via targeting critical steps of the virus replication cycle [9,10,11,12,13]. 

Quercetin is one of the most pervasive flavonoids found in plants, mostly in vegetables and fruits [14]. Quercetin has several therapeutic properties including antioxidant [15], antibacterial [16], anticancer [17], and antiviral effects [18,19]. Additionally, previous studies showed virucidal and virus replication blockade and viral entry-inhibitory activity for quercetin against influenza viruses [20,21,22] 

Hesperidin belongs to a class of flavonoids called flavanones, abundantly found in citrus fruits. Its aglycone form is called hesperetin. Hesperidin has multiple pharmaceutical activities against hyperlipidemia, diabetes, inflammation, and viruses [23]. A previous study showed that hesperidin affects the replication of the influenza viruses [24]. 

Molecular docking studies are widely used and easily applicable methods for the design of new drug candidates or the proposal of new mechanisms of action for the new or already existing drugs [25,26]. These methods are very promising to predict the binding mode and affinity of a molecule towards the binding site of a specific receptor pocket. Molecular docking achieves three important goals: binding prediction, virtual screening, and binding affinity estimation as well [27,28]. 

Several virtual and review articles discussed the potential antiviral activities of hesperidin [29], curcumin [2], and quercetin [29,30] against SARS-CoV-2. Based on the previously mentioned facts and in continuation of our research to find a potential drug candidate targeting SARS-CoV-2 [5,31,32,33,34,35,36,37], we decided to examine the three polyphenolic compounds in detail as promising anti-SARS-CoV-2 antivirals using in vitro and in silico approaches against both the spike (S) and main protease (Mpro) pockets of SARS-CoV-2. 

## 2. Results

### 2.1. Cytotoxicity of Tested Compounds

**Figure 2:**
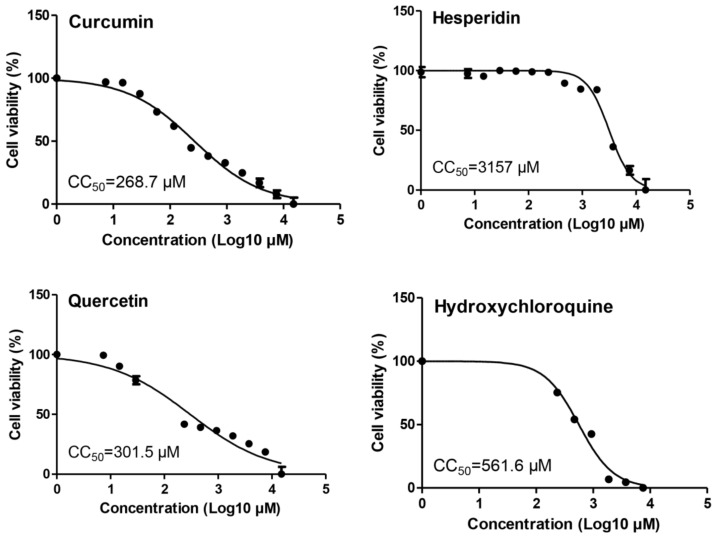
Cytotoxicity assay of the tested compounds in Vero E6 cells. The cytotoxicity of curcumin, hesperidin, quercetin, and hydroxychloroquine based on the dose–response was determined using MTT. The 50% cytotoxic concentration (CC_50_) was calculated for each compound using nonlinear regression analysis of GraphPad Prism software (version 5.01).

The cytotoxicity levels of curcumin, hesperidin, quercetin, and hydroxychloroquine in Vero E6 cells were measured by MTT assay and the result showed that the 50% cytotoxic concentration (CC_50_) values of curcumin, hesperidin, quercetin, and hydroxychloroquine were 268.7, 3157, 301.5, and 561.6 µM, respectively (Figure 2). 

### 2.2. Antiviral Activity of Tested Compounds

**Figure 3:**
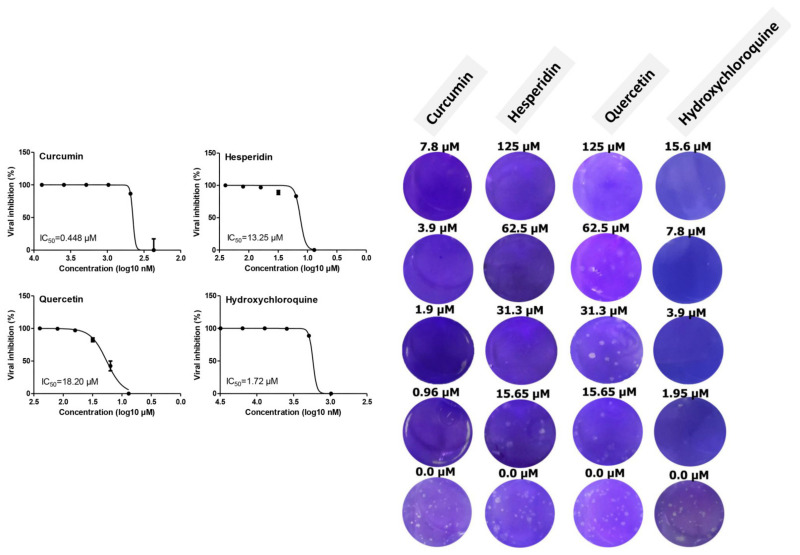
Antiviral activity of curcumin, hesperidin, quercetin, and hydroxychloroquine using a plaque reduction assay against SARS-CoV-2 virus in Vero E6 cells. Inhibitory concentration 50% (IC_50_) values were calculated using nonlinear regression analysis of GraphPad Prism software (version 5.01) by plotting log inhibitor versus normalized response (variable slope).

The antiviral activities of curcumin, hesperidin, and quercetin versus hydroxychloroquine based on the dose–response were determined using a plaque reduction assay. The result showed that the 50% inhibitory concentration (IC_50_) values of curcumin, hesperidin, quercetin, and hydroxychloroquine were 0.44, 13.25, 18.2, and 1.72 µM, respectively. Untreated infected cells showed virus-induced plaques (Figure 3). 

**Figure 4:**
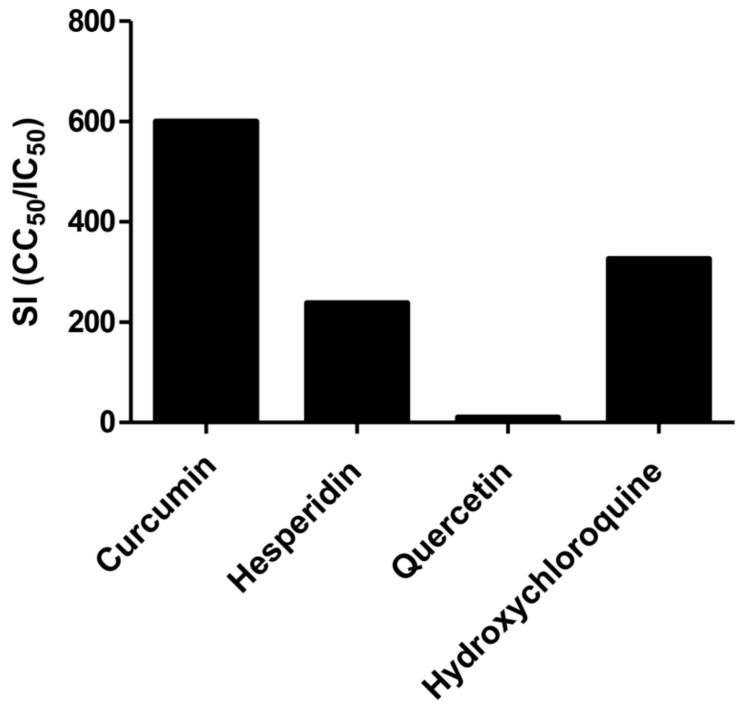
The selectivity indexes (CC_50_/IC_50_) of curcumin, hesperidin, and quercetin compared with hydroxychloroquine.

Consequently, the selectivity indexes (SI = CC_50_/IC_50_) of curcumin, hesperidin, quercetin, and hydroxychloroquine compounds showed 600, 238, 17, and 326, respectively (Figure 4). 

### 2.3. Time Course Analysis

**Figure 5:**
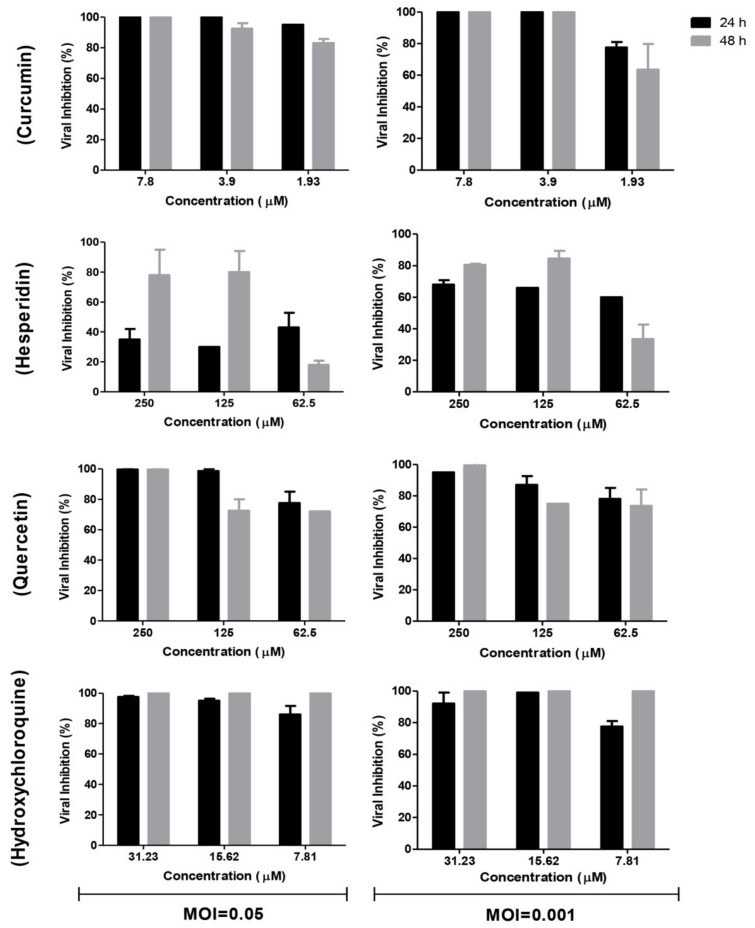
Time course analysis of antiviral effects of curcumin, hesperidin, and quercetin compared with hydroxychloroquine, calculated based on the plaque titration assay. Vero E6 cells were infected at a multiplicity of infection (MOI) of 0.05 and 0.001 of SARS-CoV-2 then treated with curcumin (7.8, 3.9, and 1.93 µM), hesperidin (250, 125, and 62.5 µM), quercetin (250, 125, and 62.5 µM), and hydroxychloroquine (31.23, 15.6, and 7.8 µM). The cells were incubated for 24 h and 48 h post-infection followed by titration using plaque infectivity assay. The viral inhibition % was calculated based on the reduction in viral count in treated and untreated virus for each compound.

**Figure 6:**
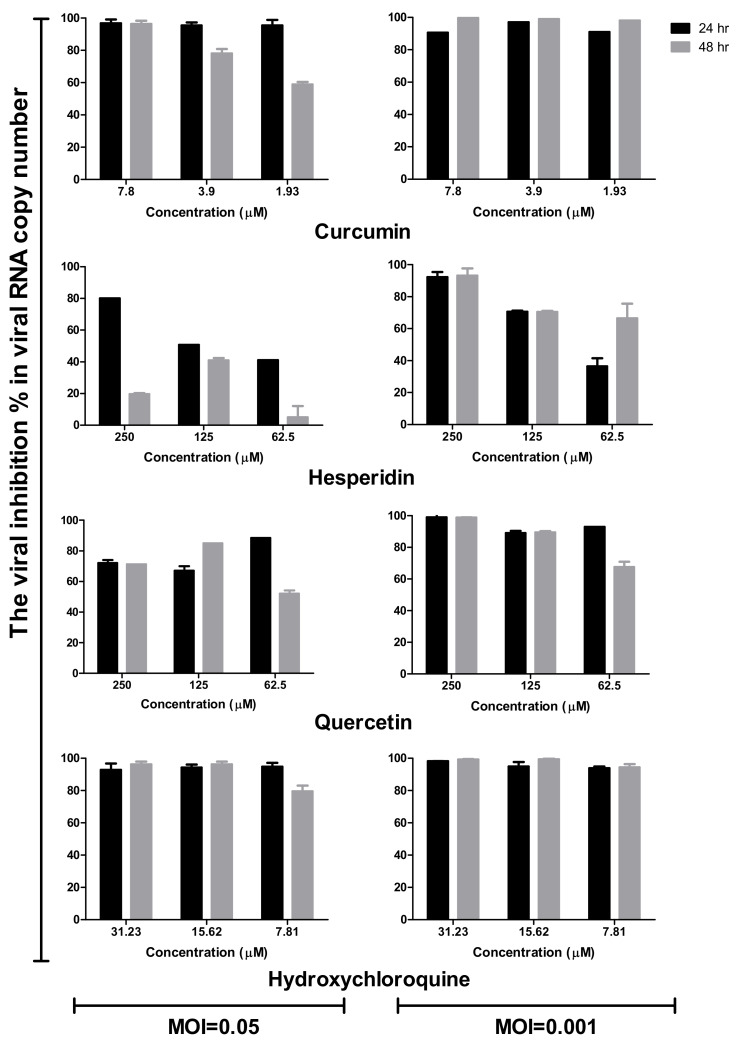
Time course analysis of antiviral effects of curcumin, hesperidin, and quercetin comparing with hydroxychloroquine based on viral RNA copy number. Vero E6 cells were infected at MOI of 0.05 and 0.001 of SARS-CoV-2 then treated with curcumin (7.8, 3.9, and 1.93 µM), hesperidin (250, 125, and 62.5 µM), quercetin (250, 125, and 62.5 µM), and hydroxychloroquine (31.23, 15.6, and 7.8 µM). The cells were incubated for 24 h and 48 h post-infection followed by detecting the viral RNA for treated/infected and untreated/infected samples using real-time RT-PCR. The relative copy number of viral RNA inhibition (%) was normalized to the virus-untreated control.

In comparison to the mock-treated and virus-infected cells at different multiplicities of infection (MOIs), the supernatants of treated and infected cells with different concentrations of tested compounds were subjected to viral quantification by plaque infectivity assay (Figure 5) and RT-qPCR (Figure 6). Viral titers were significantly reduced in the case of the addition of hydroxychloroquine at different concentrations of infected cells (Figure 5 and Figure 6). Curcumin and quercetin had strong inhibitory effects on virus replication at different MOIs and showed more than 90% reduction in plaque counts and viral RNA copy numbers. No significant difference was observed between viral inhibitory percentage at 24 h and 48 h post-treatment using curcumin and quercetin, thus indicating their stability. Different concentrations of curcumin and quercetin significantly affected the virus titers tested by plaque titration assay and real-time RT-PCR in a dose-dependent profile (Figure 4 and Figure 5). Hesperidin showed the lowest antiviral activity using several concentrations at different MOIs compared to curcumin and quercetin (Figure 5 and Figure 6). 

### 2.4. Mode of Action

**Figure 7:**
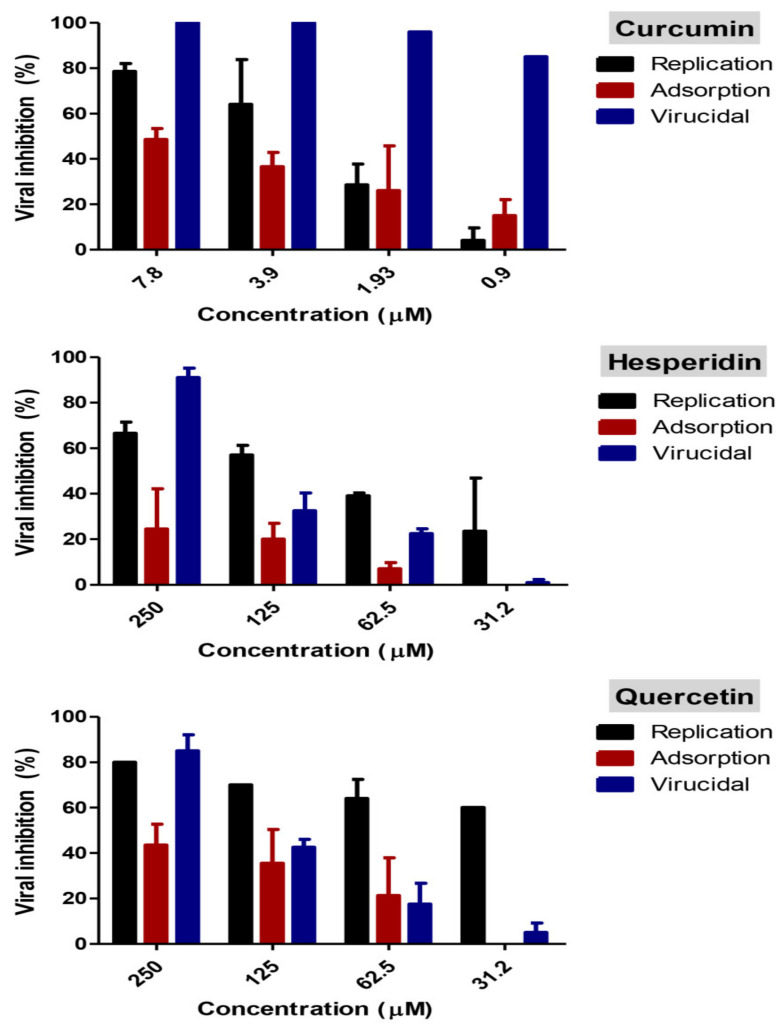
Modes of action of curcumin, hesperidin, and quercetin. Virucidal, viral replication, and viral adsorption mechanism were studied for each compound at different concentrations using a plaque reduction assay. The three tested compounds could mainly act via virucidal activity against SARS-CoV-2. The untreated virus was included in each mode of action as a virus control and viral inhibition % for each mechanism of tested compound was calculated based on the percentage of virus reduction %.

Percent inhibition for various mode of actions are shown in Figure 7. Interestingly, curcumin had a combination of viral inhibitory effect on SARS-CoV-2 at different viral stages. Curcumin has >99% virucidal effect indicating that it possibly acts directly on the virion causing inactivation. Additionally, it showed 76% and 45% inhibitory effect at 7.8 µM concentration during viral replication and adsorption stages, respectively. At a lower dose, curcumin’s virucidal effect had the most effective action compared with replication and adsorption mechanisms. Hesperidin exhibited the virucidal effect with more than a 90% viral inhibitory effect as well as an approximately 65% inhibitory effect on virus replication. A negligible reduction in viral inhibition was detected during the application of a viral adsorption mechanism. Quercetin showed virucidal effects on the virus as well as effects on viral replication. 

### 2.5. Docking Studies

The active site of SARS-CoV-2 Mpro was observed to be branched in shape with the presence of its co-crystallized native inhibitor (N3) inside it in an asymmetric manner. Analyzing the docking results of the examined three polyphenolic compounds (curcumin **1**, hesperidin **2**, and quercetin 3) was considered in the presence of one reference standard (hydroxychloroquine 4) in the case of S docking process or two reference standards (hydroxychloroquine 4 and the co-crystallized native inhibitor (N3) 5) in the case of Mpro docking process. For S docking, the binding scores of curcumin 1 and hesperidin 2 (−7.02 and −7.92 kcal/mol, respectively) were found to be better than that of hydroxychloroquine 4 (−6.60 kcal/mol). On the other hand, quercetin 3 showed the least score value (−6.48 kcal/mol). However, for Mpro docking the binding scores of curcumin 1 and hesperidin 2 (−7.28 and −8.37 kcal/mol, respectively) were found to be higher than that of hydroxychloroquine 4 (−7.05 kcal/mol), and at the same time, very close to the docked co-crystallized reference inhibitor (N3) with a binding score of −9.51 kcal/mol. Moreover, quercetin 3 got the least score value (−6.23 kcal/mol) which seems to be very close to the aforementioned values as well. Their interactions with the amino acids of the S and Mpro receptors of SARS-CoV-2 are depicted in Table 1. 

Concerning the S docking, curcumin 1 did not form any H-bonds at the receptor pocket. However, hesperidin 2 formed only one H-bond with Arg514 amino acid. Moreover, quercetin 3 achieved one H-bond with Thr445 and one pi-H interaction with Ile446 amino acids. 

Regarding the Mpro docking process, the docked co-crystallized inhibitor (N3) formed three H-bonds with His164, Cys145, and Thr26 amino acids of the receptor. However, curcumin 1 bound three amino acids (Thr26, His41, and Gln189) by hydrogen, H-pi, and pi-H bonds, respectively. On the other hand, hesperidin 2 showed the formation of three H-bonds with Gly143, Glu166, and His163 amino acids. Furthermore, quercetin 3 was stabilized inside the Mpro pocket through the formation of only one H-bond with Thr26 amino acid (Table 2 and Figures S1 and S2).

Analyzing the two previously mentioned docking processes, it is obvious that the docking results of the tested polyphenolics (**1**–**3**) on SARS-CoV-2 Mpro were found to be superior to its S docking results. This indicates their promising antiviral inhibitory effects on SARS-CoV-2 replication rather than its adsorption (penetration). 

## 3. Discussion

COVID-19 is a major global public health issue. Efforts for the screening of effective antiviral agents against SARS-CoV-2 are of major importance. Polyphenolic compounds extracted from some plants act as the main source for the development and discovery of antiviral drugs [38,39]. In the present study, we investigated the potential in vitro antiviral effects of curcumin, hesperidin, and quercetin against the newly emerging SARS-CoV-2 in Vero E6 cells. The current study showed several novel findings; our results demonstrated that curcumin, hesperidin, and quercetin have antiviral effects against SARS-CoV-2 with IC_50_ values 0.44, 13.25, and 18.2 µM, respectively. Interestingly, curcumin showed a relatively high selectivity index (SI = 600), while quercetin represented the lowest selectivity index (SI = 10). 

Previous studies have suggested that curcumin has antiviral activity through several modes of action based on the tested virus. For example, curcumin inhibited HCV, Zika, and chikungunya replication during binding and fusion stages [40,41]. Additionally, curcumin has an effect on virus replication machinery by reducing the activity of integrase of HIV-1 [42]. In line with previous studies which speculated about the direct effects of curcumin on the membrane of enveloped viruses [43], our results suggest that curcumin has virucidal effects on infectious SARS-CoV-2 in a dose-dependent fashion. We also showed that curcumin has effects on viral replication as well as adsorption mechanisms. 

Hesperidin is an old herbal medicine widely used for several pharmaceutical purposes. Haggag et al., suggested the possibility of using hesperidin for prophylaxis and the treatment of SARS-CoV-2 based on their observations in previous findings [29]. Consistent with their hypothesis, our finding showed that hesperidin has a virucidal effect as well as the effect on the viral replication mechanism. The virus-inhibitory effect of hesperidin was lower at 24 h post-treatment but well-improved at 48 h post-treatment. This is consistent with the known information about the low cellular uptake of hesperidin (0.023 ± 0.008 nmol/min/mg protein) [44], which may delay the impact of hesperidin on replication as one of the main modes of action, and hence impairing the anti-SARS-CoV-2 activity of hesperidin at early time points (24 h post-treatment, Figure 4). A previous study showed that hesperidin improves cell-autonomous immunity, which directly affects viral replication as demonstrated by our finding [24]. In a recent study assessing the anti-Hepatitis B virus (HBV) activity, quercetin and hesperidin had promising inhibitory effects on HBsAg and HBeAg expressions. Interestingly, quercetin showed high anti-HBV activity whereas hesperidin had moderate effects [45].

In accordance with Rojas and his colleagues who showed that quercetin appears to have direct and host-mediated antiviral effects against Hepatitis C virus [46], our results showed that quercetin does affect SARS-CoV-2 directly prior to infection. A previous study indicated that quercetin had inhibitory effects against influenza virus when the virus was pre-treated with it before infection [21]. The same study indicated that quercetin might exhibit antiviral activity via interaction with the hemagglutinin of influenza and subsequently interfere with virus infectivity. 

The aforementioned docking results largely confirmed the very promising binding affinities of the tested polyphenolic compounds (1, 2, and 3) towards the Mpro pocket of SARS-CoV-2 and therefore the consequently expected intrinsic activities as well. This explains the previously mentioned in vitro results concerning these very promising compounds (1, 2, and 3) as SARS-CoV-2 maturation inhibitors rather than penetration. According to these findings, we propose the tested polyphenolic compounds (curcumin 1, hesperidin 2, and quercetin 3) as potential SARS-CoV-2 Mpro inhibitors. However, the mode of action should be further confirmed using other in silico and in vitro assays. In addition, other modes of action not tested in this study may exist.

According to our findings, the tested compounds against SARS-CoV-2 could effectively act as novel antiviral agents through their virucidal effects. This needs to be validated through in vivo experiments and clinical trials. In this study, the three tested compounds were tested individually and were not evaluated for synergistic actions, thus we recommend further investigation of combinations of the three effective compounds. This may provide several advantages over using a single compound including enhancing antiviral potency, reducing drug toxicity, overcoming viral resistance, and reducing drug dose. 

## 4. Materials and Methods

### 4.1. In Vitro Virological Studies

#### 4.1.1. Virus, Cells, and Compounds

Vero E6 cells were cultured in Dulbecco’s modified Eagle’s medium (Lonza, Verviers, Belgium) supplemented with 10% fetal bovine serum (Gibco, New York, NY, USA), and 1% antibiotic–antimycotic mixture (Lonza). The cells were incubated at 37 °C in a humidified atmosphere of 5% CO_2_. An hCoV-19/Egypt/NRC-3/2020 SARS-CoV-2 virus (Accession Number on GSAID: EPI_ISL_430820) was propagated in Vero E6 cells and harvested after cytopathic effects (CPE) appearance. Viral stock was titred titrated using plaque infectivity assay and stored at −80 °C.

Curcumin (Bio Basic, Canada INC.), hesperidin (Carl Roth, Karlsruhe, Germany), and quercetin (Molekula, Molekula, United Kingdom) compounds were evaluated for their potential antiviral activity against SARS-CoV-2 virus. Hydroxychloroquine (Sanofi, Paris, France) was used as a positive control antiviral drug throughout our study. Dimethyl sulfoxide (DMSO) (Sigma-Aldrich) was used at 10% in water as a proper solvent of the three tested compounds as well as hydroxychloroquine. The prepared stock solutions were sterilized by syringe filter with 0.2 micron pore size (Millipore, Billerica, MA, USA) and stored at −20 °C. 

#### 4.1.2. In Vitro Cytotoxicity Assay

Cytotoxicity assays in Vero E6 cells of curcumin, hesperidin, quercetin, and hydroxychloroquine were evaluated in vitro using the 3-(4, 5-dimethylthiazol -2-yl)-2, 5-diphenyltetrazolium bromide (MTT) method [47] with minor modification. Briefly, Vero E6 cells were cultivated in 96-well plates with cell density ≈ 1 × 10^4^ /well and incubated for 24 hrs at 37 °C in 5% CO_2_.

On the next day, the cells were washed using 1X PBS and treated with different concentrations (15 mM–1 µM) of curcumin, hesperidin, and quercetin in triplicates. Untreated control cells were included in each plate. 

After incubating for 24 h, MTT solution (20 µL of 5 mg/ml stock solution) was added to each well and further incubated at 37 °C for 4 h followed by dissolving the formed formazan crystals using a volume of 200 µL DMSO. Absorbance of formazan solutions were measured at λmax 540 nm with 620 nm as a reference wavelength using a multi-well plate reader. Cytotoxicity percentage was determined based on the untreated cells. 

The % cytotoxicity curve of each tested compound was plotted using Graph Pad Prism 5 (Graph Pad Software Inc., San Diego, CA, USA) and the toxic concentration for 50% of the cells (CC_50_) was determined from the linear equation.

#### 4.1.3. Plaque Reduction Assay

Vero E6 cells were seeded in six-well culture plates (10^5^ cells/ml) and incubated for 24 h at 37 °C in 5% CO_2_. Previously titrated hCoV-19/Egypt/NRC-03/2020 SARS-CoV-2 virus was diluted to optimal virus dilution (10^-2^), which allowed countable plaques, and mixed with the safe concentration of each tested compound (<CC_50_). The tested compound/virus mixture was incubated for 1 h at 37 °C before being added to the cells. Growth medium was removed from the 6-well cell culture plates and virus-compound mixtures were inoculated in duplicate. Each plate included uninfected control cells (as indicator of cell viability) and untreated infected cells (as negative control). After 1 h contact time for virus adsorption, 3 mL of DMEM supplemented with 2% agarose (Lonza), 1% antibiotic–antimycotic mixture (Lonza), and 2% FBS (Lonza) were added to the cell monolayer. The plates were left to solidify and incubated at 37 °C for four days. A volume of 1 ml of 10% formalin was added to each well for 2 h for cell fixation and virus inactivation, then the over layer was removed by water. For the visualization of plaques, fixed cells were stained using 1 ml of the staining solution (1% crystal violet dissolved in 20% methanol and 80% distilled water) for 10 min, dye was discarded, plate wells were rinsed with water and dried. Untreated virus was included in each plate as a control. The percentage of viral inhibition curve of each tested compound against SARS-CoV-2 was plotted using Graph Pad Prism 5 and the 50% inhibitory concentration (IC_50_) was determined from the nonlinear regression curve-fit analysis.

#### 4.1.4. Time Course Analysis

Vero E6 cells were infected at MOIs of 0.05 and 0.001 of the virus then treated with curcumin (7.8, 3.9, and 1.93 µM), hesperidin (250, 125, and 62.5 µM), quercetin (250, 125, and 62.5 µM), and hydroxychloroquine (31.23, 15.6, and 7.8 µM), respectively. The cells were incubated for 24 and 48 h post-infection at 37 °C in 5% CO_2_ in infection medium. Mock-infected cells without treatment in the same plate were used as control. Cell culture supernatants were collected at each point of the time course infection in order to perform viral quantification by plaque infectivity assay and RT-qPCR [48]. Percentage of viral reduction in plaques counts and viral RNA copy numbers in comparison to control wells was recorded as follows:

(1) $$\%\;\mathrm{Inhibition}=\frac{Viral\;count\;\left(untreated\right)\;-\;Viral\;count\;\left(treated\right)}{Viral\;count\;\left(untreated\right)}\times 100$$

#### 4.1.5. Study of the Mode of Action

##### Adsorption Mechanism

The viral adsorption mode of action was tested according to Zhang et al. [49], with minor modifications. Briefly, Vero E6 cells were cultivated in a six-well plate and incubated at 37 °C for 24 h. The cells were washed using 1X PBS then curcumin (7.8, 3.9, 1.9, 0.96, 0 µM), hesperidin (250, 125, 62.5, 31.3, 0 µM), and quercetin (250, 125, 62.5, 31.3, 0 µM) were directly applied to cells and incubated for 1 h. Non-absorbed materials were removed by washing cells using 1X PBS. The virus was added to the pretreated cells then incubated for 1 h, and then 3 ml DMEM supplemented with 2% agarose and 1% antibiotic–antimycotic mixture was added. Uninfected control cells were included in each plate to determine cell viability. Plates were left to solidify and were then incubated at 37 °C for 4 days to allow for the formation of viral plaques. The plaques were fixed and stained as described above to calculate the percentage reduction in plaque formation compared to control virus wells.

##### Replication Mechanism

The effect of each compound on the viral replication mechanism was studied according to a published protocol [50] with minor modifications. Briefly, Vero E6 cells were cultivated in a six-well plate and incubated at 37 °C for 24 h. The cells were infected with the virus then incubated for 1 h at 37 °C for virus infection. The cells were washed using 1X PBS to remove non-adsorbed virus. Curcumin (7.8, 3.9, 1.9, 0.96, 0 µM), hesperidin (250, 125, 62.5, 31.3, 0 µM), and quercetin (250, 125, 62.5, 31.3, 0 µM) were added to each well of infected cells, and after 1 h contact time, 3 ml of over layer medium of DMEM supplemented with 2% agarose and 1% antibiotic–antimycotic mixture was added to the cell monolayer. Uninfected control cells were included in each plate. Plates were incubated for three days then were fixed using 10% formalin solution for 1 h and stained with crystal violet. The percentages of viral inhibition were calculated based on untreated virus control wells.

##### Virucidal Mechanism

The virucidal mode of action was tested as previously shown [51]. In a six-well plate, Vero E6 cells were cultured a day before infection and incubated at 37 °C. On the next day, 120 µL of serum-free DMEM containing diluted virus was added to curcumin (7.8, 3.9, 1.93, 0 µM), hesperidin (250, 125, 62.5, 31.3, 0 µM), and quercetin (250, 125, 62.5, 31.3, 0 µM), and incubated for 1 h at room temperature. Uninfected control cells were included in each plate. After incubation, the mixture was added to the Vero E6 cell monolayer. After 1 h contact time, a DMEM overlayer medium was added to the cell monolayer and then incubated for three days. The viral inhibition of each concentration of each compound was calculated as previously described.

### 4.2. Docking Studies

The aforementioned three polyphenolic compounds were examined against both the S and Mpro receptors of SARS-CoV-2 in two different docking processes to confirm the proposed mechanism of action as Mpro inhibitors based on their promising in vitro findings towards viral replication rather than adsorption through molecular docking studies using the MOE 2019.012 suite [52]. They were examined in comparison to both hydroxychloroquine (**4**)—used as a standard reference during the in vitro studies—and also the protein co-crystallized native inhibitor (N3, **5**) to investigate the binding interactions and modes. 

#### 4.2.1. Preparation of the Polyphenolic Compounds (**1**–**3**) and the References (**4** and **5**)

The 3D chemical structures of curcumin **1**, hesperidin **2**, quercetin **3**, and hydroxychloroquine **4** were downloaded from the Drug Bank database website. They were prepared for the docking process according to the previously described steps [53]. Furthermore, the co-crystallized native inhibitor (N3, **5**) was extracted from the downloaded protein (6LU7) [54] and saved together with the aforementioned compounds (**1**, **2**, **3**, and **4**) in one database file to be uploaded during the corresponding docking process.

#### 4.2.2. Preparation of the S and Mpro Target Receptors of SARS-CoV-2

Both S and Mpro receptors of SARS-CoV-2 were downloaded from the Protein Data Bank website (PDB code: 6VW1 [55] and 6LU7 [54], respectively). Then, the downloaded X-ray protein structures were subjected to the full preparation steps described earlier [56]. Moreover, the Site Finder tool was applied to choose and isolate the largest pocket in case of S and Mpro proteins—which was found to be the same binding pocket of the native co-crystallized N3 inhibitor in case of the later—as dummy atoms for the docking processes [57,58]. 

#### 4.2.3. Docking of the Polyphenolics to the S and Mpro Receptors of SARS-CoV-2

A general docking process for each one of the previously prepared databases containing the tested polyphenolic compounds (1, 2, and 3) together with one reference standard (4) in S docking and two reference standards (4 and 5) in Mpro docking was applied. The largest pocket was selected to be the docking site and the default docking methodology following the previously described program specifications was performed for each docking process [59]. After completion of the two docking processes, the obtained poses were studied well and one pose for each tested compound was selected based on its better binding score inside the receptor pocket and RMSD_refine value as well for each process. 

## 5. Conclusions

In conclusion, three polyphenolic compounds (curcumin 1, hesperidin 2, and quercetin 3) were examined in vitro against SARS-CoV-2 and showed very promising inhibitory results compared to hydroxychloroquine as a reference standard. Additionally, their MOA study revealed their potential activity targeting the viral maturation cycle rather than its penetration. Therefore, two different molecular docking processes for the aforementioned compounds (**1**–**3**) were carried out against both the S and Mpro pockets of SARS-CoV-2 to examine their recommended possible MOA. It is worth mentioning that their docking results on the Mpro receptor of SARS-CoV-2 were very promising and a little bit superior to the corresponding S docking results. Therefore, this indicates their potential inhibitory activity on the viral replication process rather than its penetration (adsorption). These findings appeared to be very interesting for further examining these compounds through preclinical and clinical studies either alone or in combination with each other to obtain a therapeutic combating SARS-CoV-2 effectively in the near future. 

## Figures and Tables

**Table 1:** The binding scores and amino acid interactions of the tested three polyphenolic compounds (**1**–**3**) compared to hydroxychloroquine (**4**) and the docked N3 inhibitor (**5**) inside the binding pocket of SARS-CoV-2 Mpro.

No.	Polyphenolics and Controls	R ^a^	S ^b^	RMSD_Refine ^c^	Amino Acid Interactions	Bond Distance (A)
**1**	Curcumin	S	−7.02	0.68	−	−
		Mpro	−7.28	1.16	Thr26/ H-donor His41/H-pi Gln189/pi-H	2.82 3.70 4.50
**2**	Hesperidin	S	−7.92	1.99	Arg514/H-acceptor	3.13
		Mpro	−8.37	2.00	Gly143/H-acceptor Glu166/H-donor His163/H-acceptor	2.91 3.06 3.29
**3**	Quercetin	S	−6.48	1.69	Thr445/H-donor Ile446/pi-H	3.19 4.27
		Mpro	−6.23	1.17	Thr26/H-donor	3.07
**4**	Hydroxychloroquine	S	−6.60	1.98	His345/H-acceptor Arg518/pi-H Arg518/pi-H	3.10 3.48 4.74
		Mpro	−7.05	1.91	His163/H-acceptor His41/H-pi	3.51 3.90
**5**	N3 (docked)	Mpro	−9.51	1.65	His164/H-donor Cys145/H-donor Thr26/H-donor	3.11 3.37 3.63

^a^ R: The receptor pockets of SARS-CoV-2 (Spike (S) and main protease (Mpro)). ^b^ S: The score of a ligand inside the binding pocket (Kcal/mol), ^c^ RMSD_refine: The root mean squared deviation between the predicted pose and the crystal structure.

**Table 2:** 3D receptor binding pictures showing the interactions and positioning of the tested three polyphenolic compounds (**1**–**3**) compared to the docked N3 inhibitor (**5**) inside the binding pocket of SARS-CoV-2 Mpro.

Tested Comp.	R	3D Interactions	3D Positioning
Curcumin (1)	S	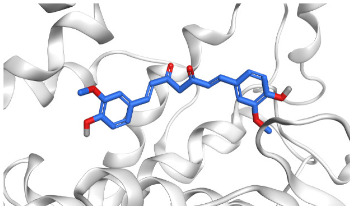	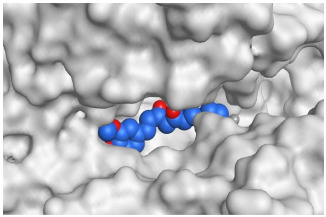
	Mpro	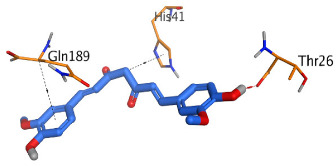	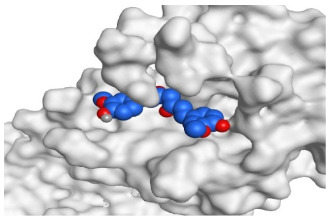
Hesperidin (2)	S	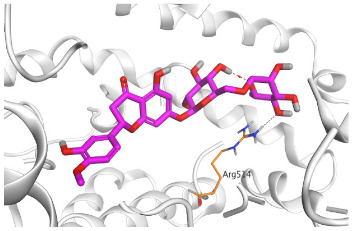	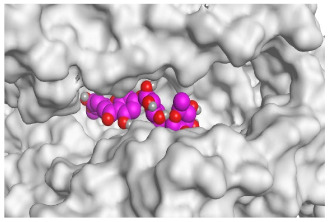
	Mpro	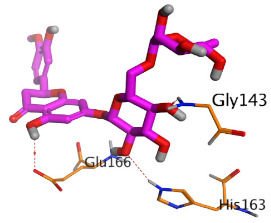	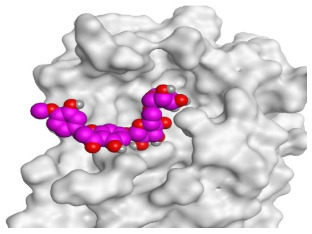
Quercetin (3)	S	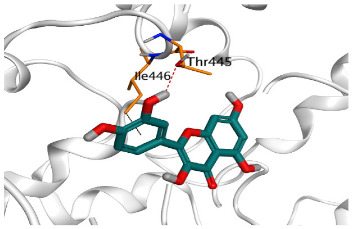	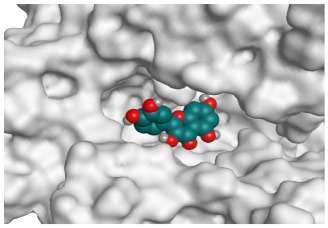
	Mpro	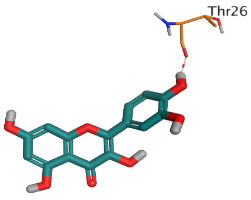	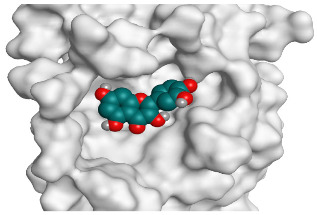
Docked N3 (5)	Mpro	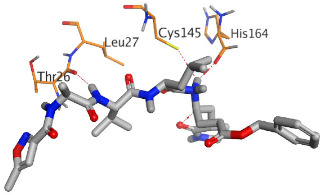	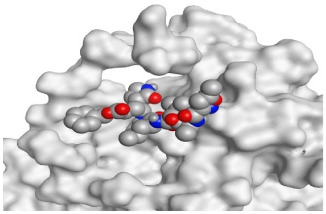

Red dash represents H-bonds and black dash represents H–pi interactions.

## Data Availability

The data presented in this study are available within the article and Supplementary Materials.

## Supplementary Materials

The following are available online at https://www.mdpi.com/article/10.3390/pathogens10060758/s1, Figure S1: 2D pictures representing the binding interactions of the tested three polyphenolic compounds (**1**–**3**) compared to hydroxychloroquine (**4**) and the docked N3 inhibitor (**5**) inside the binding pocket of SARS-CoV-2 Mpro, Figure S2: 3D receptor binding pictures showing the interactions and positioning of hydroxychloroquine (**4**) inside the binding pocket of SARS-CoV-2 Mpro.
