# Supplementary material for: Bioactive Polyphenolic Compounds Showing Strong Antiviral Activities against Severe Acute Respiratory Syndrome Coronavirus 2
Source: Pathogens. 2021 Jun 15;10(6):758. doi: 10.3390/pathogens10060758 (PMC8232731; doi:10.3390/pathogens10060758)
Supplement: Supplementary file 1 [file pathogens-10-00758-s001.zip › pathogens-1176518-supplementary.pdf]

Supplementary data

<table>
<tr><th>No.</th><th>R</th><th>2 D binding interactions</th></tr>
<tr><td rowspan="2">1</td><td>S</td><td>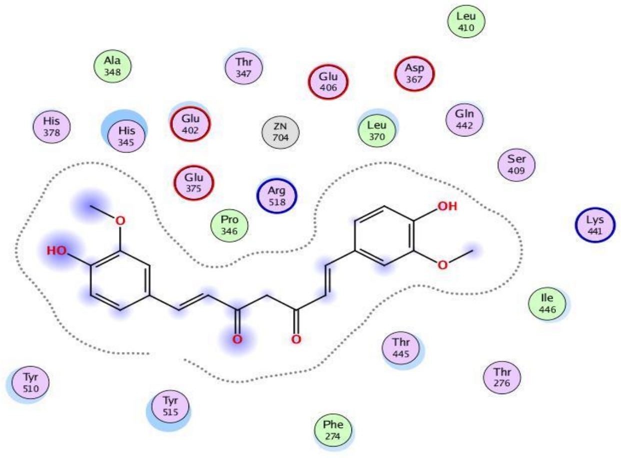
</td></tr>
<tr><td>Mpro</td><td>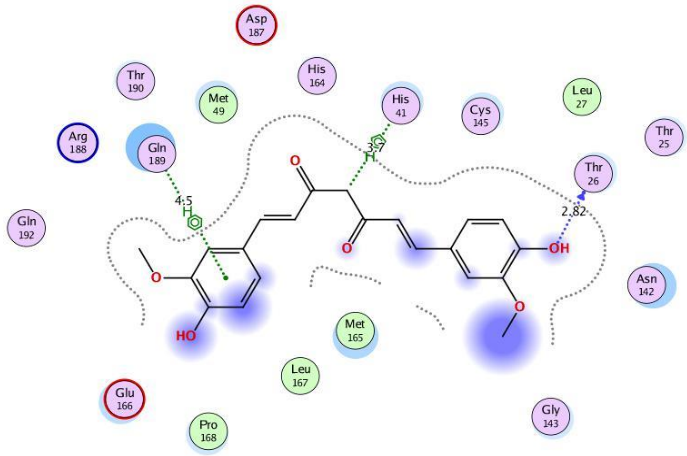
</td></tr>
<tr><td>2</td><td>S</td><td>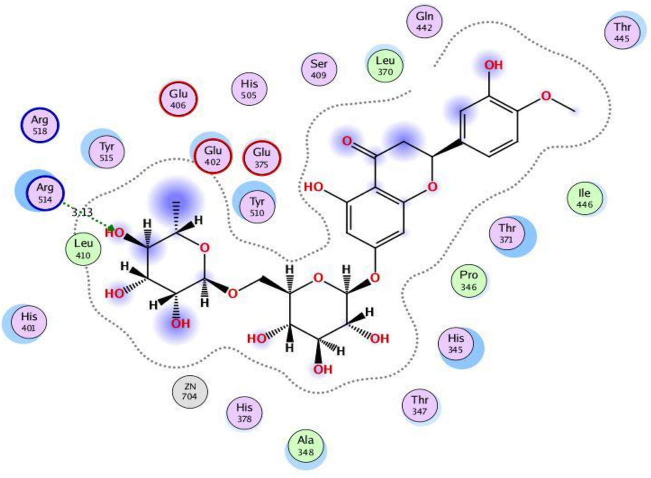
</td></tr>
</table>

	Mpro	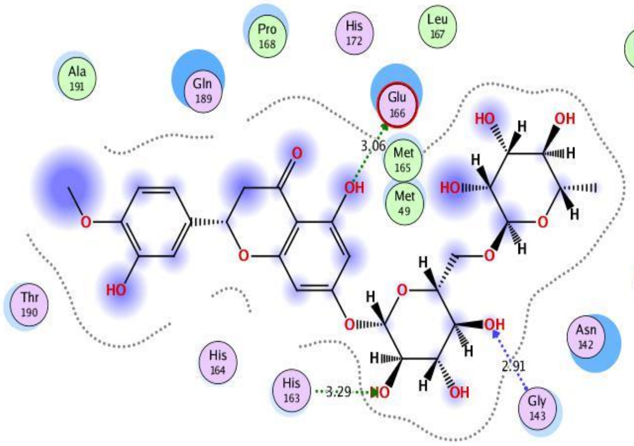Ala 191<br>Gln 189<br>Pro 168<br>His 172<br>Leu 167<br>Leu 27<br>Thr 25<br>Glu 166<br>Met 165<br>Met 49<br>His 41<br>Thr 26<br>Asn 142<br>Gly 143<br>Thr 190<br>His 164<br>His 163<br>3.06<br>3.29<br>2.91
3	S	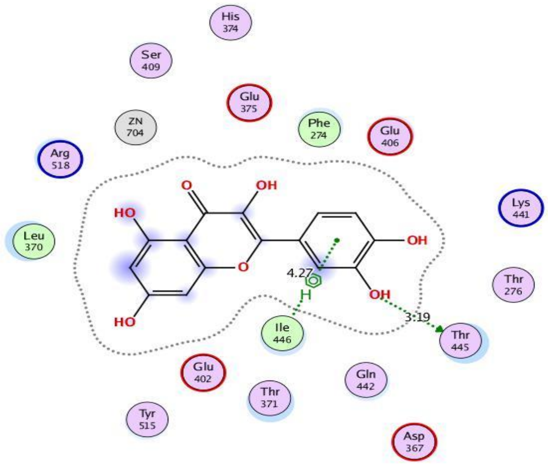His 374<br>Ser 409<br>Glu 375<br>ZN 704<br>Phe 274<br>Glu 406<br>Arg 518<br>Lys 441<br>Leu 370<br>Thr 276<br>Ile 446<br>Thr 445<br>Glu 402<br>Thr 371<br>Gln 442<br>Tyr 515<br>Asp 367<br>4.27<br>3.19
	Mpro	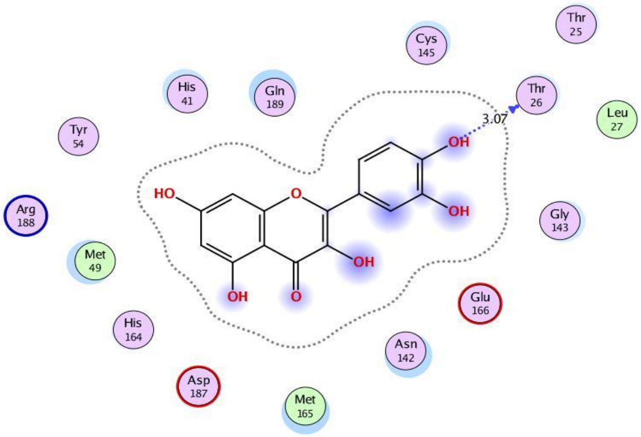Thr 25<br>Cys 145<br>His 41<br>Gln 189<br>Thr 26<br>Leu 27<br>Tyr 54<br>Arg 188<br>Gly 143<br>Met 49<br>Glu 166<br>His 164<br>Asn 142<br>Asp 187<br>Met 165<br>3.07

4	S	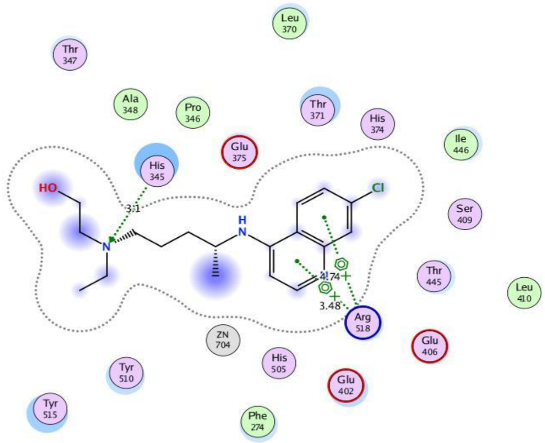
	Mpro	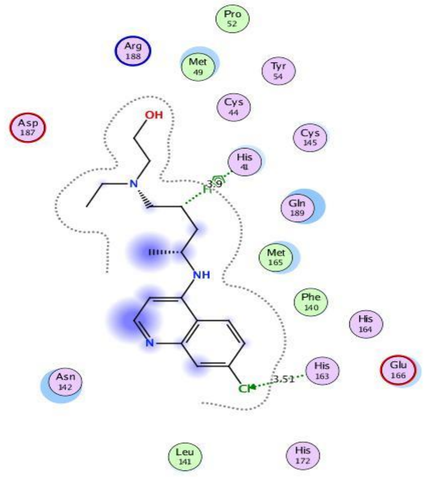
5	Mpro	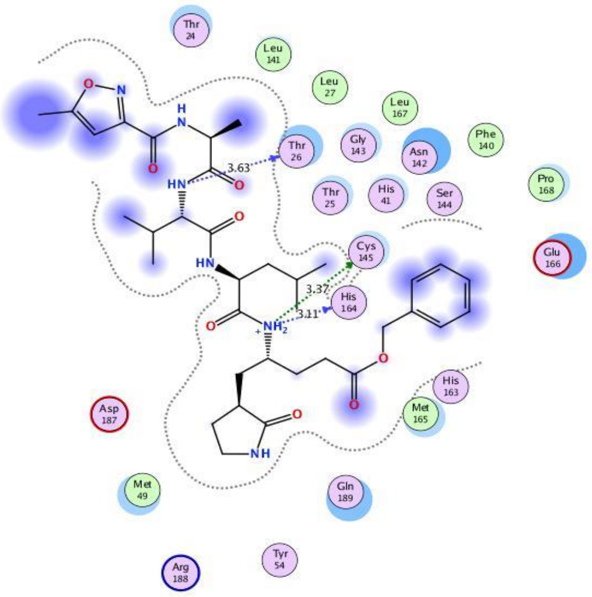

Figure S1. 2D pictures representing the binding interactions of the tested three polyphenolic compounds (**1–3**) compared to hydroxychloroquine (**4**) and the docked N3 inhibitor (**5**) inside the S and Mpro pockets of SARS-CoV-2.

Tested comp.	R	3 D interactions	3 D positioning
Hydroxychloro-quine (4)	S	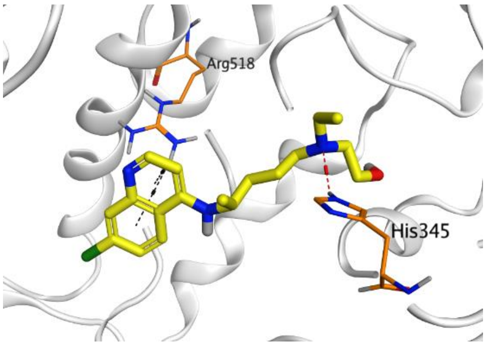	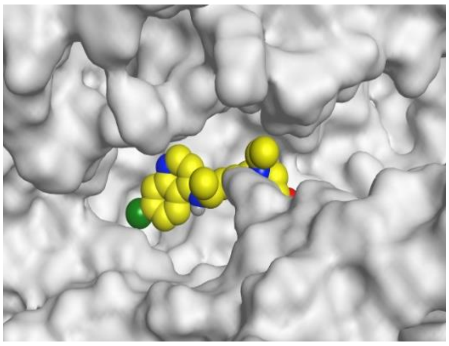
	Mpro	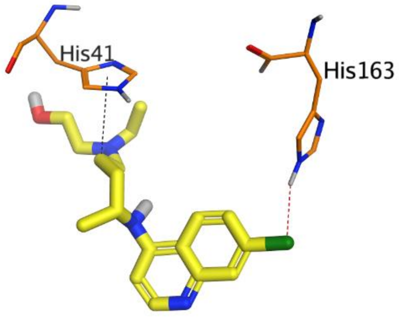	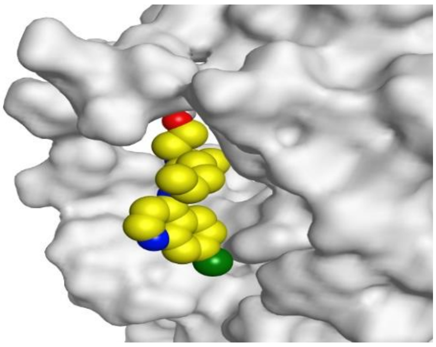

Figure S2. 3D receptor binding pictures showing the interactions and positioning of hydroxychloroquine (4) inside the S and Mpro pockets of SARS-CoV-2. Red dash represents H-bonds and black dash represents H-pi interactions.
